# Infantile Spasms, Clinical Manifestations of a Rare Brain Tumor: A Case Report and Literature Review 

**DOI:** 10.22037/ijcn.v16i4.34911

**Published:** 2022-03-14

**Authors:** Rezvan HOSSEINZADEH, Amin TAVALLAII, Ehsan KEYKHOSRAVI, Morteza BEHNAMFAR, Mahnaz BANAEI, Meisam BABAEI

**Affiliations:** 1 Student Research Committee, School of Medicine, Babol University of Medical Sciences, Babol, Iran; 2 Neurosurgery Department, Mashhad University of Medical Sciences, Mashhad, Iran; 3 Student Research Committee, School of Medicine, North Khorasan University of Medical Sciences, Bojnurd, Iran; 4 Hazrat Ali Asghar Children’s Hospital, Iran University of Medical Sciences, Tehran, Iran; 5 Department of Pediatrics, North Khorasan University of Medical Sciences, Bojnurd, Iran

**Keywords:** Desmoplastic Infantile Ganglioglioma, Brain Tumor, Infantile Spasms

## Abstract

Desmoplastic infantile ganglioglioma (DIG) has a favorable prognosis and is classified as a benign infantile brain tumor. The DIG is more common in children under 2 years of age than in other age groups. This report introduces a 5.5 month-old infant who was referred with infantile spasms and diagnosed with a brain tumor. Brain magnetic resonance imaging showed a large heterogeneous mass in the right hemisphere with shifting to the other side. The patient underwent surgery. The extra-axial mass was completely resected, and the diagnosis of DIG grade I was confirmed by pathology. After one year, patient development was normal, and the seizures did not recur. In addition, the general condition was good. With a brief review and search in the literature, 13 case reports were identified 9 of which were male cases. The mean age of initial manifestation to final tumor diagnosis was 4 months. Out of 13 patients, 8 cases were reported with the mass origin in the right hemisphere. The most commonly observed tumors were glioma (n=4) and hypothalamic hamartoma (n=3). Except for three patients who died, the remaining had a complete recovery after tumor removal with a seizure-free interval at follow-up.

## Introduction

 Epileptic spasms are regarded as a known type of seizure occurring with an incidence of 25 per 100,000 live births in the United States and Western Europe. An underlying cause can be determined in approximately 75% of patients. The etiologic classification in infantile spasms has been recommended as genetic, structural/metabolic, or unknown causes. Structural causes, such as brain tumors, are among uncommon etiologic factors that probably clinically present with infantile spasms (1).

 Desmoplastic infantile ganglioglioma (DIG) is a rare infantile brain tumor that rarely appears with infantile spasms. The DIG (also called desmoplastic infantile astrocytoma of infancy) is primarily observed in infants and young children, with a male predominance. Patients with DIGs typically present in infancy with macrocephaly or partial complex seizures. The median reported age of presentation is about 5 months. The involvement of multiple lobes is common with a predilection for the frontal and parietal lobes (2). 

 Vandenberg was the first who described DIG in 1987 (3). The DIG is an uncommon intracranial tumor that, despite the massive size and severe desmoplasia with astrocytic and ganglionic cells, is benign and can occur in an infant at the age of fewer than 18 months. The result of the DIG tumor's surgical resection has been successful to date (2,4).

 Most of the present studies have reported that infantile spasms have appeared with central nervous system diseases; however, the occurrence of infantile spasms with brain tumors is not common (4). In this report, we present a rare case of infantile brain tumor that clinically presents with infantile spasms and have an overview on similar cases reported in the literature. 

## Case Report

The patient, a 5.5-month-old infantile male, was born after an uneventful full-term pregnancy and a natural vaginal delivery. His birth weight and head circumference were 3200 g and 34 cm, respectively. The family history included a father with thalassemia major and a cousin treated for hydrocephalus. He was referred to the hospital with recurrent epileptic spasm attacks, mainly while waking up, lasting less than 20 seconds. His attacks started 2 weeks before the referral. The spasm was extension type with nystagmoid eye movements. 

During the physical examination, he was conscious and had attention to his surroundings. He did fix and follow. On evolutionary examination, he comfortably held his neck and rolled over. Eyes function and limb strength were normal. Reflexes were asymmetric with exaggerated reflexes in the left lower limb and mild left hemiparesis. Moreover, no skin lesions were observed.

**Fig. 1:**
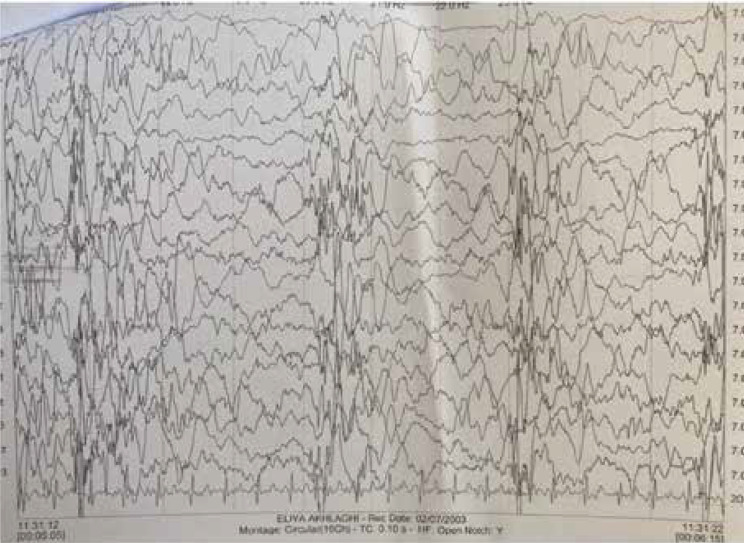
**B** **efore** **S** **urj** **u** **ry**, this EEG shows Burst suppression pattern and modified hypsarrhythmia. also some degree of asymmetry between two hemisphere. (EEG characteristics: bipolar montage, Amp: 70, HF: 60; LF: 0.1; Speed 3)

**Figure 2:**
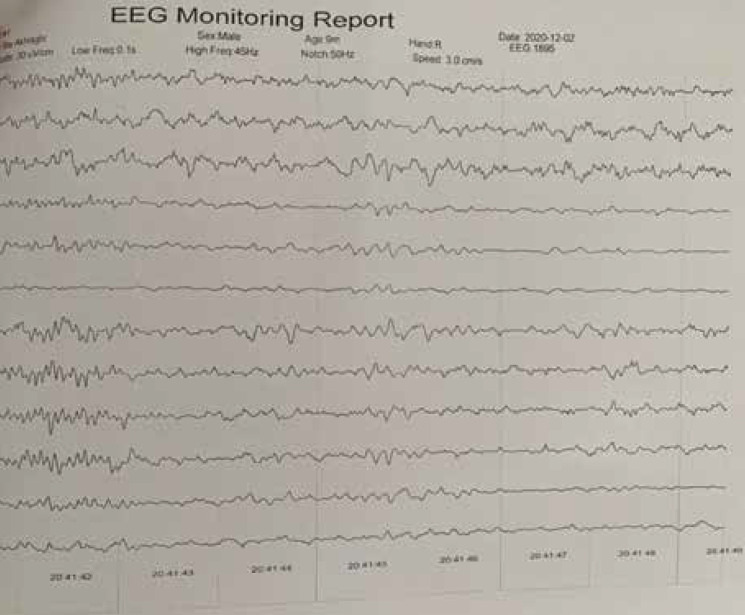
**A** **fter** **S** **urjury**, this EEG shows normal background without hypps pattern, and some mild asymmetry. (EEG characteristics: bipolar montage, Amp; 30, HF; 45, LF; 0.1, Speed; 3)

An electroencephalogram (EEG) showed a burst suppression pattern, indicating modified hypsarrhythmia and disorganized background with mild asymmetry between the two hemispheres (figures 1 and 2). The patient underwent adrenocorticotropic hormone treatment and was asked for brain magnetic resonance imaging (MRI). 

**Figure 3:**
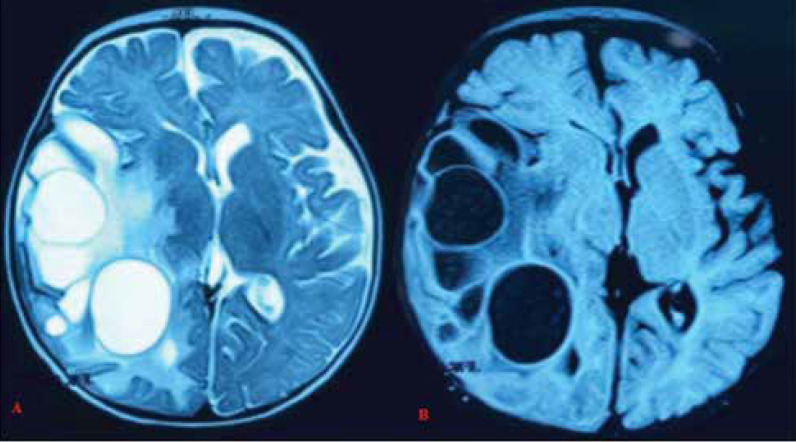
(A) Axial T2W MRI image, (B) Axial FLAIR MRI image, before tumor removal

The brain MRI of the patient exhibited large heterogeneous multilobulated and cystic masses in the right temporoparietal region with shifting to the left (Figure 3). After injection, a wide, irregular, and heterogeneous enhancement was observed in the subdural space in the right parietal area, and a brief enhancement was observed in the left subdural space. Cystic tumoral lesions in this area were presented as a differential diagnosis; therefore, a biopsy was necessary. A consultation with a neurosurgeon was requested. 

**Figure 4:**
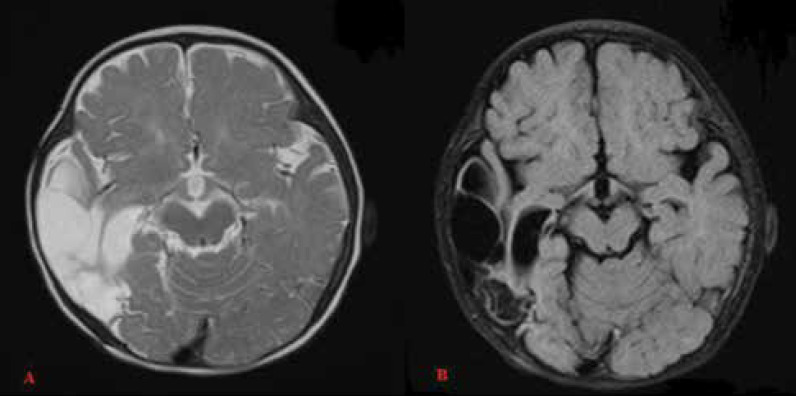
(A) Axial T2W MRI image, (B) Axial FLAIR MRI image, after tumor removal

The patient underwent surgery (Figure 4). The extra-axial mass was completely resected, and the diagnosis of DIG grade I was confirmed based on the pathologic result. Additionally, vimentin protein and glial fibrillary acidic protein were positive in pathology tests. At present, the patient is only on phenobarbital, and the seizures do not recur. Furthermore, the general condition is good.

## Discussion

One of the most critical seizures in infancy is an infantile spasm. Infantile colic is one of the differential diagnoses of infantile spasms. Additionally, infantile spasms will have a worse prognosis if it is not diagnosed early and treated. The causes of infantile spasms are symptomatic (80%), cryptogenic, and idiopathic. Common symptomatic causes include neonatal ischemic hypoxic encephalopathy and tuberous sclerosis, which do not have a favorable prognosis. Less common causes include genetic and acquired structural disorders. Brain tumors are regarded as a structural and acquired disorder clinically presenting as infantile spasms (5).

According to the Google search for the words brain tumor and infantile spasms, 13 patients have been reported. The onset of symptoms varied from 1 to 14 months, and the mean age of onset of symptoms was 4 months, which is approximately consistent with the age of onset of symptoms in the present patient (i.e., 5.5 months) (6-13). According to gender, the patients included nine male and four female cases (6-13). The mean age of tumor diagnosis from the onset of symptoms to diagnosis was within 2 weeks to 2 months, which was 2 weeks in the present case. Except for three patients, the duration of diagnosis for each of them was 9 (11), 18 (12), and 21 (13) months. 

Of 13 patients, 8 patients were reported with the origin of the mass in the right hemisphere. The origin of the mass in three patients was the left hemisphere (7, 9, 10). In one patient, the mass was in the hypothalamus (6). In another patient, the mass was in the posterior fossa (13). In the present case, the source of the mass was the right hemisphere, compatible with the most common sites. Pathologically, the most commonly observed tumors were glioma (n=4) (8, 10, 11) and hypothalamus hamartoma (n=3) (6, 7, 9). 

In addition, in the present case, the final pathology of DIG was reported. Except for one patient, all patients had hypsarrhythmia and modified hypsarrhythmia in the EEG. Moreover, as a common electrophysiologic finding, modified hypsarrhythmia was also reported in this case (figures 3 and 4). 

Except for three patients who died (the first case from glioma grade III [11], the second case from medulloepithelioma [13], and the third case from ganglioglioma [8]) and one patient with primitive neuroectodermal tumor (12) who suffered from psychomotor regression, the remaining patients had a complete recovery with complete control of seizures after the surgical resection of the mass. The present case also had complete seizure control within 9 months of follow-up after tumor removal, and her development was normal.

This study received no specific grant from any funding agency in the public, commercial, or not-for-profit sectors. 

## Author’s Contribution

All authors had full access to the data, contributed to the study, approved the final version of the manuscript, and took responsibility for its accuracy and integrity. 

## Conflicts of Interest

The authors have no conflict of interest in preparing and submitting this report.

## References

[1] J. Eric Pina-Garza, Kaitlin C. James, chapter 1 (2019). Fenichel’s Clinical Pediatric Neurology. A signs and symptoms approach.

[2] Charles Raybaud, Zoltan Patay, James Barkovich ( 2018). Pediatric Neuroimging.

[3] Kamoun S, Azouz H, Zemmali M, Haouet S, Kchir N (2019). Desmoplastic infantile ganglioglioma. Pan Afr Med J.

[4] Alexiou GA, Stefanaki K, Sfakianos G, Prodromou N ( 2008). Desmoplastic Infantile Ganglioglioma. Pediatr Neurosurg.

[5] Swaiman KF, Ashwal S, Frriero DM, Sohor NF, Finkel RS, Gropman AL (2017). Swaiman's pediatric neurology. principles and practice.

[6] Asanuma H, Wakai S, Tanaka T, Chiba S (1995). Brain tumors associated with infantile spasms. Pediatr Neurol.

[7] Fox J, Hussain S, Sankar R, Kerrigan JF (2018). Hypothalamic Hamartoma With Infantile Spasms: Case Report With Surgical Treatment. Semin Pediatr Neurol,.

[8] Gabriel YH (1980). Unilateral hemispheric ganglioglioma with infantile spasms. Ann Neurol,.

[9] Işık U, Saltık S, Tanrıkulu B, Özek MM (2017). Hypothalamic hamartoma presenting with infantile spasms. Child’s Nerv Syst,.

[10] Kotagal P, Cohen BH, Hahn JF (1995). Infantile spasms in a child with brain tumor: Seizure-free outcome after resection. J Epilepsy,.

[11] Ruggieri V, Caraballo R, Fejerman N (1989). Intracranial tumors and West syndrome. Pediatr Neurol,.

[12] Viani F, Romeo A, Mastrangelo M, Asi H (1994). Infantile Spasms Secondary to the Surgical Excision. J Child Neurol,.

[13] Aktan G, Şimsek A, Aysun S (1997). Brainstem Tumor With Infantile Spasms. J Child Neurol,.

